# Medical Society of the State of New York

**Published:** 1901-11

**Authors:** F. C. Curtis

**Affiliations:** Secretary. Albany


### MEDICAL SOCIETY OF THE STATE OF NEW YORK.

BACK VOLUMES OF THE TRANSACTIONS.

*Editor Buffalo Medical Journal:*

Sir—The society has in its possession a surplus of its annual volumes for certain years past beyond its current needs, and it appears desirable to place them at the disposal of the individual members. The years thus available, some more fully than others, are: reprints of the years 1807—31 in one volume; reprints of the years 1840-43; and volumes from 1868 to 1898 inclusive; also for the years 1861 and 1866.

These or any part of them are offered gratuitously so long as they last, to members and delegates of the society, and, if not speedily called for by them, to all members of the County Medical Societies; they will also be given on application to society or other libraries. The only expense will be for packing and transportation, which is estimated will be about $2.00 for the whole number offered; this may be sent with the request and they will be forwarded prepaid.

Some volumes are also available for the years 1891, 1892, 1895, 1896 and 1898, at 50 cents a volume; those of years subsequent to 1898 can be had at $1.00 a volume.

F. C. CURTIS, Secretary.

Albany, September 1, 1901.

